# Validation and performance of a rapid HIV-1 viral load assay

**DOI:** 10.1128/spectrum.02646-26

**Published:** 2026-09-23

**Authors:** Katharine Uhteg, Mohammad H. Bayan, Alec Boudreau, Zoe O. Demko, Robin J. MacGowan, Kevin P. Delaney, Yukari C. Manabe, Matthew M. Hamill, Heba H. Mostafa

**Affiliations:** 1 Johns Hopkins Hospital Medical Microbiology Laboratory, Baltimore, Maryland, USA; 2 Division of Infectious Disease, Johns Hopkins School of Medicine 229385 , Baltimore, Maryland, USA; 3 Centers for Disease Control and Prevention 1242, Atlanta, Georgia, USA; 4 Division of Medical Microbiology, Department of Pathology, Johns Hopkins School of Medicine 1500 , Baltimore, Maryland, USA; Barnard College, Columbia University, New York, New York, USA

**Keywords:** HIV, diagnostics, point of care (POC), quantitative tests, NAT

## Abstract

Current diagnostic algorithms for HIV-1 primarily depend on antigen-antibody tests, with nucleic acid tests reserved for confirmation and monitoring of antiretroviral therapy. Incorporating a quantitative nucleic acid test at the point of care could provide faster diagnosis and viral load measurement in a single clinical encounter, expediting linkage to care. This study evaluates the Xpert HIV-1 Viral Load XC (Xpert VL XC) assay as a candidate for primary nucleic acid testing use within HIV-1 diagnostic workflows, compared to standard-of-care approaches. The Xpert VL XC demonstrated a measurable analytical range of 60–1 × 10^6^ copies/mL (*R*^2^ = 0.99) with 100% detection across tested concentrations. Precision analyses showed greatest variability at the lowest concentration, with acceptable assay reproducibility. Agreement analyses using 20 HIV-1 positive and 10 HIV-1 negative plasma samples showed 100% agreement compared to the Roche cobas HIV-1 (quantitative) assay. The Xpert VL XC was compared to four other HIV assays, including the Xpert HIV-1 Qual XC. The Xpert VL XC produced 3.2% invalid results and achieved negative agreement of ≥96.8%. Positive agreement ranged from 80.8% to 90.0%, with most discrepancies occurring below the 40 copies/mL lower limit of quantification threshold. The Xpert HIV-1 Qual XC was the only assay that detected all 26 samples that were positive using the standard immunoassay diagnostic algorithm. Average turnaround time was 3 h, faster than the 21 h average for conventional batch testing. These findings highlight the limitations of implementing rapid molecular HIV-1 diagnostics and the need for both sensitive and rapid approaches.

**IMPORTANCE:**

Current testing algorithms for the diagnosis of HIV-1 initially test samples with an antigen/antibody test followed by an antibody differentiation test if the screening test is positive. Nucleic acid tests are used to aid in the diagnosis (qualitative and quantitative assays) or monitoring a patient’s response to antiretroviral therapy (quantitative). However, a quantitative diagnostic nucleic acid test may have additional utility, particularly in point-of-care settings, or near the patient care site. This would allow both diagnosis and initial viral load measurement to occur during a single visit, permitting rapid linkage to care. We evaluated the Xpert HIV-1 Viral Load XC test as a potential tool for primary nucleic acid testing within the HIV-1 diagnostic algorithm and assessed its suitability for use under the unique constraints of point-of-care environments.

## INTRODUCTION

There are an estimated 1.2 million people in the United States living with HIV (1). HIV exists as two types, with HIV-1 being the most prevalent in the United States, accounting for over 99% of cases (2). In the United States, laboratory diagnosis has been based on an algorithm of antigen/antibody (Ag/Ab) screening followed by confirmation and differentiation between HIV-1 and HIV-2 via antibody testing. In the screening algorithm, nucleic acid detection is used to resolve discordant and indeterminate results, such as in cases where the samples are antigen positive but differentiation by antibody detection is indeterminate. Using molecular methods as the first step in the diagnostic algorithm has been recommended only for pre-exposure prophylaxis (PrEP) in individuals treated with long-acting cabotegravir, an injectable drug formulation approved for use in the prevention of HIV transmission by the US Food and Drug Administration (FDA) (3). Diagnosing HIV can be particularly challenging in these individuals because once infected, the amount of HIV ribonucleic acid (RNA) can be very low and does not raise an antibody response for many months (4). Hence, the use of a highly sensitive molecular assay that can detect both RNA and pro-viral DNA in POC settings would be beneficial for individuals evaluated for HIV pre- and post-exposure prophylaxis (PrEP and PEP) or those with injectable antiretroviral therapy (ART) exposure (3). Additionally, viral load monitoring is integral for managing persons with HIV-1 and is used before and after initiating ART and after ART regimen switches. Accurate POC quantification of viral load may allow for same-day feedback to the patient (5).

Commercial molecular qualitative, quantitative/diagnostic, and quantitative HIV-1 nucleic acid tests (NATs) are available, but few are FDA-approved for diagnostic use; to date, there are no waived NATs that can be used at the POC to provide results within a single clinical encounter (6). The FDA-approved HIV-1 NATs have varying degrees of automation, physical footprint, and throughput; however, they are all run on large, highly complex platforms. POC diagnostic molecular assays that are simple to use and have a more rapid turnaround would be an ideal solution to facilitate linkage to care during the same clinical visit. In this study, we evaluated the performance of a quantitative NAT assay, currently not approved or cleared by the FDA, yet available outside the United States, is CE-marked, and can potentially be performed as a POC, sample-to-answer test: the Xpert HIV-1 Viral Load XC test (Xpert VL XC, Cepheid, Sunnyvale, CA, USA). The assay’s performance was compared to the Roche cobas HIV-1 quantitative assay (Roche, Indianapolis, IN, USA) in addition to the Roche cobas HIV-1/2 qualitative and the Xpert HIV-1 Qual XC test (Xpert Qual XC). Finally, all assays were compared with the standard-of-care antigen/antibody-based screening algorithm.

## MATERIALS AND METHODS

### Materials for the analytical evaluation

HIV-1 quantified viral stock was purchased from EXACT Diagnostics (Bio-Rad, Hercules, CA, USA). The original concentration of the EXACT control was 2 × 10^6^ copies/mL, measured by droplet digital polymerase chain reaction (ddPCR). Serial dilutions were made in normal human plasma (NHP) (LGC Clinical Diagnostics, Middlesex, UK) to generate concentrations ranging from 1 × 10^6^ to 60 copies/mL. Stock dilutions of spiked samples were stored at 4°C.

### Test methods

We evaluated the Xpert VL XC assay for analytical measurable range, reproducibility, and agreement. Patient plasma or spiked samples were centrifuged for 5 min at 2,500 RPM. After spinning, 1 mL of patient plasma or a spiked sample was pipetted into an Xpert VL XC cartridge and then analyzed on the GeneXpert system using the Xpert VL XC software version 5, with a run time of 91 min.

**Fig 1:**
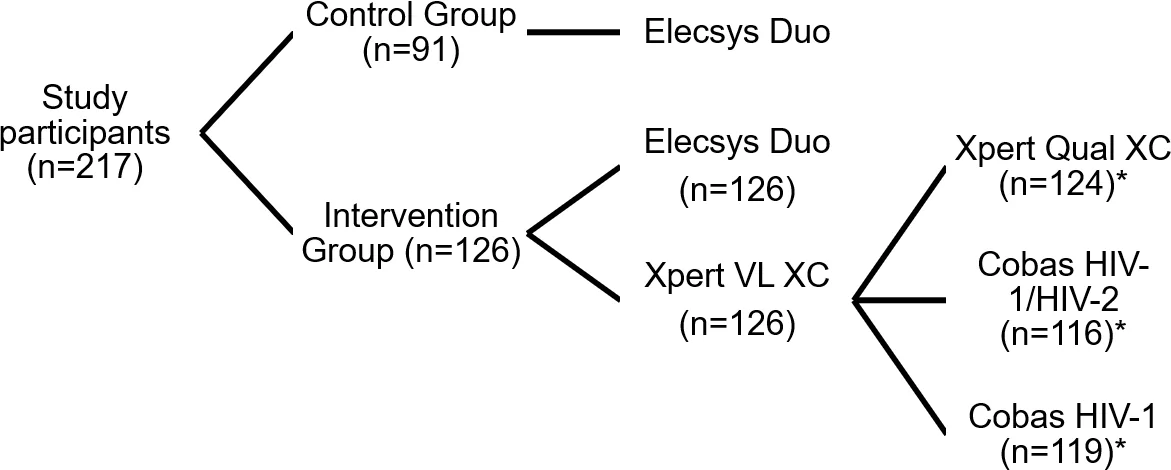
Study participants. All 126 intervention group participants received a test with the Elecsys Duo and Xpert VL XC; subsequent testing was performed on the Xpert Qual XC, cobas HIV-1/HIV-2, and cobas HIV-1 viral load assays. *Difference from 126 tested with Xpert VL XC due to insufficient quantity to test with all five assays.

In addition to the Xpert VL XC, the samples were tested on four additional assays, which included the Elecsys HIV Duo antigen-antibody test (Roche Diagnostics, Indianapolis, IN, USA), and three NATs: (i) cobas HIV-1 (Roche Diagnostics), (ii) cobas HIV-1/HIV-2 (Roche Diagnostics), and (iii) Xpert HIV-1 Qual XC (Cepheid) (Fig. 1). The Elecsys HIV Duo assay uses 60 µL of serum on the cobas e801 analyzer for a run time of 27 min. The cobas HIV-1 (quantitative) and cobas HIV-1/HIV-2 (qualitative) assays both use 650 µL of plasma and are run on the cobas 6800 system, with run times of <3.5 h each, depending on batch size. The Xpert Qual XC was run on the GeneXpert system using 100 µL of whole blood.

### Analytical performance

#### Analytical measurable range (AMR)

The Xpert VL XC package insert reports a measurable range of 40–1 × 10^6^ copies/mL, standardized against the HIV-1 standard from the World Health Organization. The measurable range was verified by running serial dilutions of spiked NHP. The control was diluted 10-fold in NHP at eight concentrations ranging from 60 to 1 × 10^6^ copies/mL. Testing was performed in replicates of 3–9, with more replicates being tested at the high (1 × 10^6^ copies/mL), mid (1 × 10^3^ copies/mL), and low (60 copies/mL) concentrations. The AMR was determined by linear analysis of scatter plots. Of note, the limit of detection (LOD) is defined in the package insert as 13.6 copies/mL, with a 95% confidence interval of 11.7–15.6 copies/mL.

#### Precision

Precision was determined by testing 3 replicates of spiked samples at three concentrations (1 x 10^6^, 1 x 10^3^, and 60 copies/mL) as well as unspiked (negative) NHP within 1 day. In addition, six additional replicates were tested over two additional days at the same concentrations. Results include the replicates tested on the same day, for a total of nine replicates at each concentration over 3 days.

#### Agreement

Plasma from 20 individuals with HIV-1 and 10 individuals without HIV-1 were run with both the Xpert VL XC and cobas HIV-1 assays. Negative samples were run the same day on both platforms. Positive samples were run first on the cobas 6800 then frozen at −80°C until later thawed and run on the GeneXpert system. Positive samples were selected at viral loads that span the analytical measurement range.

### Clinical evaluation

All aspects of this evaluation described above were reviewed and approved by the Johns Hopkins University Institutional Review Board. Additionally, the evaluation of the Xpert VL XC was performed as part of a clinical study that aimed to evaluate the role of POC HIV-1 viral load testing in facilitating earlier initiation of linkage to HIV ART or PrEP (IRB00274090) (7, 8).

Samples from a sub-group of participants from the randomized clinical trial (Fig. 1; Table S1) included a control group (*n* = 91) of participants at risk for HIV (not taking PrEP), or living with HIV (not on ART or not adherent to ART) who received the standard of care (SOC) HIV diagnostic algorithm (Elecsys HIV Duo PT as the initial screening test followed by the Geenius HIV1/2 supplemental assay (Bio-Rad) for confirmation and differentiation of antibodies to HIV-1 and HIV-2). The intervention group (*n* = 126) received the Xpert VL XC test in addition to standard antigen-antibody testing. Representative demographic, medical, and behavioral characteristics of the participants who donated samples to this analysis have been previously described (8). The 126 participants enrolled in the intervention group were also tested with at least 1 of the following tests: cobas HIV-1 quantitative (*n* = 119), cobas HIV-1/HIV-2 qualitative (*n* = 116), and Xpert Qual XC (*n* = 124) (Fig. 1). The qualitative cobas assay was run on stored frozen plasma. The rest of the assays were performed prospectively on fresh blood.

## RESULTS

### Analytical measurable range

**Fig 2:**
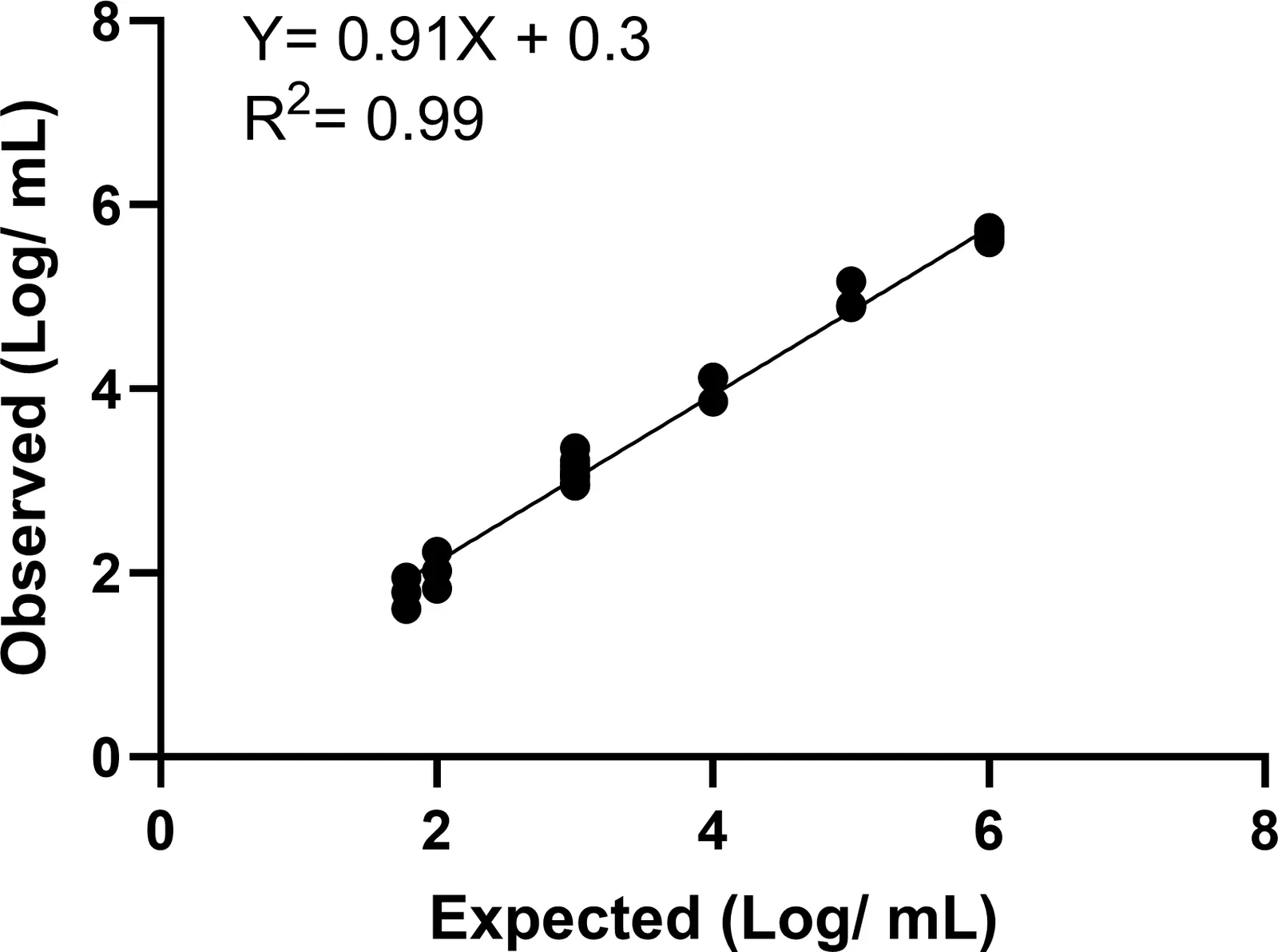
Verified measurable range of the Xpert VL XC (*n* = 30).

Replicates tested with the Xpert VL XC test at concentrations between 60 and 1 × 10^6^ copies/mL were detected at 100%. AMR (Fig. 2) was verified as the range between 60 and 1 × 10^6^ copies/mL (*R*^2^ = 0.99).

### Precision

Results of precision are shown in Table 1. Variability was greatest at the lowest tested concentration, 60 copies/mL, with a percent coefficient of variation (%CV) of 0.8–7.7% for same-day testing and 6.5% over three separate days. Three replicates were reported as <40 copies/mL and were not included in the %CV calculations.

**TABLE 1:** Precision of the Xpert VL XC assay^*a*^

	Same day		Over three days	
Expected concentration (copies/mL)	Confidence interval (95%)	%CV	Confidence interval (95%)	%CV
6 log_10_	5.5–5.8	0.3–1.1	5.6–5.8	0.9
3 log_10_	2.8–3.5	1.4–3.7	2.6–3.3	4.1
1.8 log_10_	1.5–2.1	0.8–7.7	1.5–2.0	6.5

^*a*^ CV, coefficient of variation; mL, milliliter.

### Agreement

**Fig 3:**
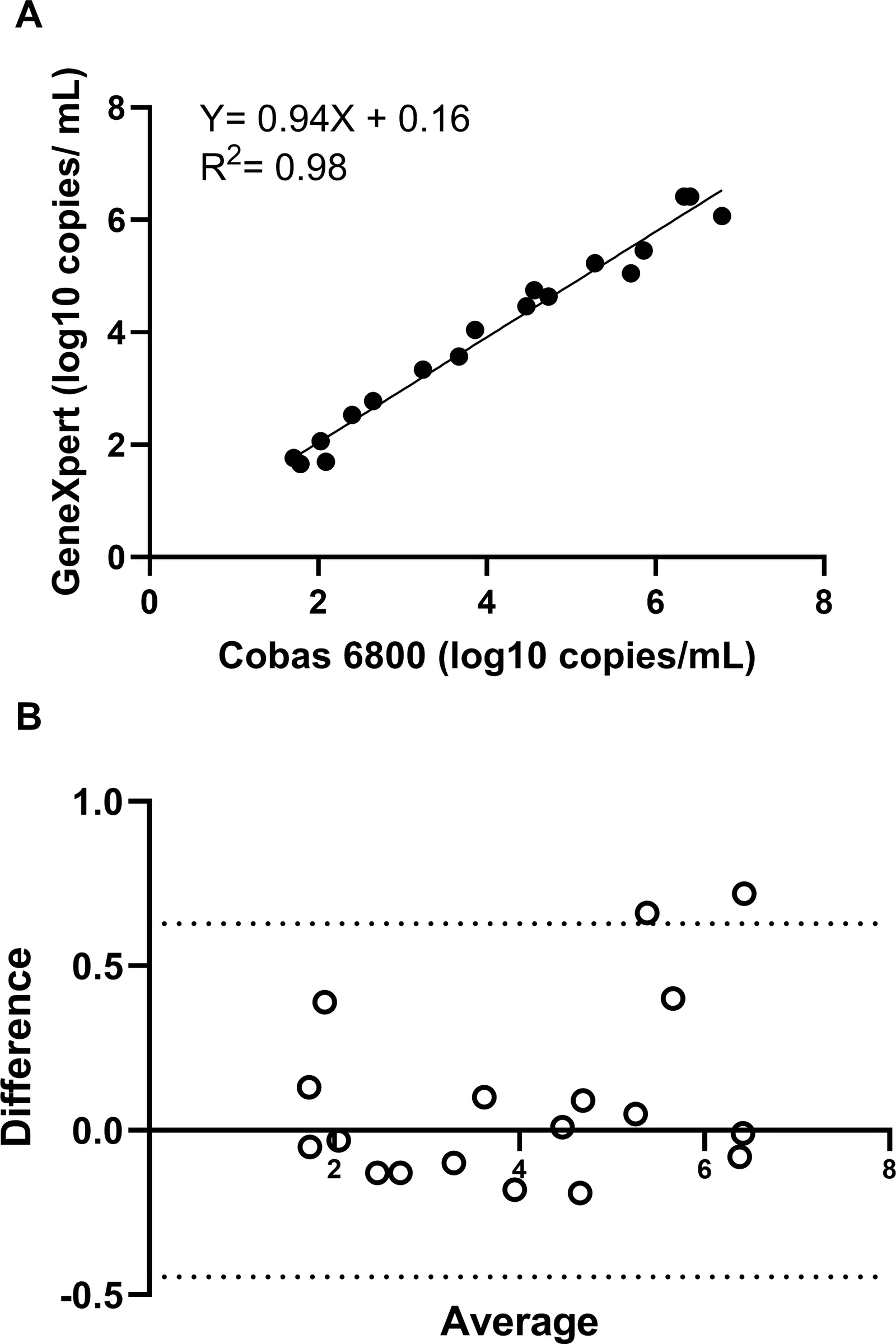
Comparison of individual patient samples, positive for HIV-1, run with both the cobas HIV-1 and Xpert VL XC assays (*n* = 18). (**A**) Regression analysis. (**B**) Bland-Altman analysis. Difference estimated as the Cobas HIV-1 VL result minus the Xpert VL XC result; larger positive values indicate lower quantity results on the Xpert assay, including two samples outside the 95% confidence interval for the difference.

A 100% positive and negative agreement was observed for samples tested by both the Xpert VL XC and the Roche cobas HIV-1 quantitative assays (Fig. 3). Two positive samples were excluded from quantitative agreement calculations due to the inability to quantify the Xpert VL XC values (below the lower limit of quantification). On average, Xpert VL XC viral load was about 0.09 log_10_ copies/mL lower than cobas HIV-1, with two samples (10%) falling outside of the 95% confidence interval.

### Clinical evaluation

A total of 126 samples from the study intervention group (Fig. 1) were tested with both the Elecsys Duo and the Xpert VL XC. Due to insufficient sample quantity, only 114 out of 126 samples were tested with all five assays. HIV-1 test results from the Xpert VL XC consisted of 21 detected, 101 not detected, and 4 (3.2%) invalid results; invalid is defined by the manufacturer as the Internal Quantitative Standard (IQS)-high and/or IQS-low Cts are not within valid range, the sample was not properly processed, or PCR was inhibited. Of the 21 samples where HIV-1 was detected, 14 had viral loads above 40 copies/mL. Of the 114 samples that were run with all five tests, and after invalid results were excluded (Xpert VL XC [*n* = 4] and Xpert Qual XC [*n* = 4]), 109 samples had valid results for all assays (Table 2). Positive and negative agreement between the Xpert VL XC assay and each of the four other tests was calculated (Table 3). The Xpert Qual XC and Elecsys Duo tests showed 100% negative agreement. The cobas HIV-1/HIV-2 (Qual) and HIV-1 (Quant) assays showed 96.8% and 98.9% negative agreement, respectively, with all discrepant Xpert VL XC samples being detected at <40 copies/mL on the respective cobas assay. Percent positive agreement was 80.8% (Xpert Qual XC, Elecsys Duo), 83.3% (cobas HIV-1), and 90.0% (cobas HIV-1/HIV-2). The Xpert VL XC assay did not detect five samples which were detected/reactive by the Xpert Qual XC and Elecsys Duo assays. Two samples with cycle thresholds of 38.6 and 39.3 by cobas HIV-1/HIV-2 had undetected HIV-1 RNA with the Xpert VL XC. Four discrepant samples with the cobas HIV-1 assay included one with a concentration of 27.3 copies/mL and three which were considered detected but <20 copies/mL, all of which were below the limit of quantification for the Xpert VL XC assay (40 copies/mL). The Xpert VL XC assay failed to detect five samples that were positive by the Elecsys Duo screening test. Of these, three were from participants with very low viral loads, reflected by high Ct values, or viral loads below the lower limit of quantification of the Roche HIV-1 assay (Table 4).

**TABLE 2:** Result comparison of the 109 samples with valid results tested with all five HIV assays

Xpert VL XC	Xpert Qual XC	cobas HIV-1/HIV-2	cobas HIV-1	Elecsys Duo	Total
RNA detected	RNA detected	RNA detected	RNA detected	Reactive	18
RNA detected	RNA detected	RNA detected	RNA not detected	Reactive	1
RNA detected	RNA detected	RNA not detected	RNA not detected	Reactive	2
RNA not detected	RNA detected	RNA detected	RNA detected	Reactive	1
RNA not detected	RNA detected	RNA detected	RNA not detected	Reactive	1
RNA not detected	RNA detected	RNA not detected	RNA detected	Reactive	1
RNA not detected	RNA detected	RNA not detected	RNA not detected	Reactive	2
RNA not detected	RNA not detected	RNA not detected	RNA not detected	Nonreactive	81
RNA not detected	RNA not detected	RNA detected	RNA not detected	Nonreactive	1
RNA not detected	RNA not detected	RNA not detected	RNA detected	Nonreactive	1

**TABLE 3:** Positive and negative agreement of the Xpert VL XC assay with the Xpert Qual XC, cobas HIV-1, cobas HIV-1/HIV-2, and Elecsys Duo assays

	Percent agreement (confidence interval)			
Agreement	Xpert Qual XC	cobas HIV-1/HIV-2	cobas HIV-1	Elecsys Duo
Positive	80.8% (60.7–93.5%) (*n* = 26)	90.0% (68.3–98.8%) (*n* = 20)	83.3% (62.6–95.3%) (*n* = 24)	80.8% (60.7–93.5%) (*n* = 26)
Negative	100.0% (96.0–100%) (*n* = 91)	96.8% (91.0–99.3%) (*n* = 94)	98.9% (94.1–100%) (*n* = 92)	100.0% (96.0–100%) (*n* = 91)
Total valid results	117	114	116	117
Excluded results^*a*^	7	2	3	4

^*a*^ Results excluded due to error or invalid in either the Xpert VL XC assay or the comparator assay.

**TABLE 4:** Discrepant results between the Xpert VL XC assay and Elecsys Duo^*a*^^,*b*^

Elecsys Duo	cobas HIV-1/HIV-2	cobas HIV-1	Xpert Qual XC	Xpert VL XC
Reactive	Ct: 39.3	<Titer (<20 c/mL)	RNA detected	RNA not detected
Reactive	RNA not detected	RNA not detected	RNA detected	RNA not detected
Reactive	RNA not detected	<Titer (<20 c/mL)	RNA detected	RNA not detected
Reactive	RNA not detected	<Titer (<20 c/mL)	RNA detected	RNA not detected
Reactive	RNA not detected	RNA not detected	RNA detected	RNA not detected

^*a*^ Limit of quantification for the cobas HIV-1 assay is 20 copies/mL.

^*b*^ Three of the discrepant samples had cobas HIV-1 positive results below the lower limit of quantification. Ct, cycle threshold.

### Turnaround time

Turnaround time, defined as the time from blood draw to result, was calculated for 102 Xpert VL XC samples. Eleven samples were excluded from calculations due to unconfirmed collection times or testing in batches outside of the workflow of the study. The turnaround time for the 102 samples was an average of 3 h and 13 min (SD, 1 h and 4 min), as compared to the average of 21 h and 6 min (SD, 18 h and 28 min) for the batch run of the in-house, viral load assay (cobas HIV-1, *n* = 126).

## DISCUSSION

The Xpert VL XC shows good performance with regards to reproducibility and specificity. With a limit of detection of 13.6 copies/mL, it falls in line with other HIV-1 quantitative assays on the market; for example, cobas HIV-1 (13.2 copies/mL), Aptima HIV-1 Quant Dx (12 copies/mL), Versant HIV-1 (50 copies/mL), Alinity m HIV-1 (20 copies/mL), and Abbott RealTime HIV-1 (40 copies/mL). Other studies that compared the Xpert VL XC assay to HIV-1 assays from Abbott, bioMérieux, Roche, and Siemens showed comparable results (8–12). Notably, the Xpert VL XC requires 1 mL of plasma, while the other assays mentioned above require between 0.2 and 0.7 mL.

Comparison of the Xpert VL XC with four other HIV assays showed agreement ranging from 96.8% and 100.0% for samples that did not contain detectable HIV-1, and from 80.8% to 90.0% for samples that tested positive for HIV-1 by the comparator assay. Five samples that were positive with the screening Elecsys Duo assay were negative with the Xpert VL XC assay, three of which had low relative viral loads (as indicated by high Ct values) or had quantities that were lower than the limit of quantification of the Roche HIV-1 assay. All five samples were detected by the Xpert Qual XC, presumably because of cell-associated viral nucleic acid (the Xpert Qual XC uses whole blood). The potential additional sensitivity of the qualitative test may be leveraged for the detection of HIV in those with low levels of viremia once integration has occurred (e.g., detection of HIV seroconversion in those on PrEP, potentially including those on long-acting injectables such as cabotegravir). Previously, this group has described that the provision of a qualitative laboratory-based HIV VL result was associated with a twofold greater linkage to care in US-based people with HIV (8). It is unclear if this association was driven by the knowledge of the viral load copy number, the presence of any detectable HIV, or for other reasons unrelated to test results. Additional studies on samples collected from patients with acute HIV are required to confirm the suggested increase in sensitivity.

FDA-approved diagnostic HIV-1 RNA tests with a viral load claim include the Aptima HIV-1 Quant Dx (Hologic) and the Alinity m HIV-1 assay (Abbott Molecular); however, both tests are highly complex and run on platforms with large footprints that would not be suitable for POC settings. Furthermore, these NATs are approved for the testing of plasma or serum only, which require centrifugation prior to running, making them inappropriate for POC testing. This is also an issue with the Xpert VL XC assay. Conversely, the Xpert Qual XC test uses whole blood, obviating the centrifugation step. Other considerations for adoption at the POC include the number of reagents as well as temperature requirements, where room temperature storage is easier to accommodate than refrigerated or frozen supplies. Another necessity would be random-access ability, decreasing the time between collection and test, and the overall time to result. The random-access feature also allows for running the user’s chosen external quality control and potential for implementation of an individualized quality control plan, whereas automated platforms may require specific controls to be run with every batch of samples, which may increase cost. Finally, the almost 1.5- to 3-h turnaround time may well exceed the willingness to wait threshold for many patients—assays that can result in under 30 min are likely needed to have an impact at POC (13–16) in clinical settings in the United States.

Our study had some limitations. Because the HIV qualitative test requires whole fresh blood, this test was compared to others that were performed on frozen, thawed plasma, though we do not expect material differences between fresh and frozen, thawed plasma for HIV molecular assays. The sample size was small, particularly the positive sample cohort, which reflected in positive agreement confidence intervals.

Our data show promising potential for POC diagnostic HIV-1 viral load assays; however, the analytical sensitivity needs to be considered if a future algorithm would rely on this assay for diagnosis. The Xpert Qual XC test may be an alternative that should be further researched. Future prospective studies in the United States might evaluate the impact of POC tests on clinical outcomes, such as same-day initiation of ART or PrEP, retention in care, or virological suppression using POC HIV-1 molecular assays with different LODs and turnaround times compared with laboratory-based reference assays.

## Data Availability

Raw data files are submitted as supplemental materials (Data set S1).

## SUPPLEMENTAL MATERIAL

Data set S1 spectrum.02646-26-s0001.xlsx Raw data of all samples tested as a part of the study.

Table S1 spectrum.02646-26-s0002.xlsx Metadata of patients tested as a part of the study.

ASM does not own the copyrights to Supplemental Material that may be linked to, or accessed through, an article. The authors have granted ASM a non-exclusive, world-wide license to publish the Supplemental Material files. Please contact the corresponding author directly for reuse.
